# Tetra­aqua­(2,2′-bipyridine-κ^2^ *N*,*N*′)manganese(II) di-μ-aqua-bis­[aqua­(2,2′-bipyridine-κ^2^ *N*,*N*′)(5-sulfonatoisophthalato-κ*O*)manganate(II)] tetra­hydrate

**DOI:** 10.1107/S1600536808020540

**Published:** 2008-07-09

**Authors:** Bing-Yu Zhang, Jing-Jing Nie, Duan-Jun Xu

**Affiliations:** a Department of Chemistry, Zhejiang University, People’s Republic of China

## Abstract

The crystal structure of the title salt, [Mn(C_10_H_8_N_2_)(H_2_O)_4_][Mn_2_(C_8_H_3_O_7_S)_2_(C_10_H_8_N_2_)_2_(H_2_O)_4_]·4H_2_O, consists of mononuclear manganese(II) cations, dinuclear manganate(II) dianions and uncoordinated water mol­ecules. The dianion is located about an inversion center; the Mn^II^ atom is coordinated by a 2,2′-bipyridine ligand, a sulfonatoisophthalate group, a water mol­ecule along with two bridging water mol­ecules in an octa­hedral geometry. The cation lies on a twofold rotation axis; the Mn^II^ atom is coordinated by four water mol­ecules and a chelating 2,2′-bipyridine ligand in a distorted octa­hedral geometry. A partially overlapped arrangement between the bipyridine ligands and the aromatic ring of the sulfoisophthalate group of adjacent anions is observed; the distance (3.357 Å) indicates π–π stacking. Hydrogen bonds, with the water mol­ecules serving as hydrogen-bond donors, lead to a three-dimensional network architecture.

## Related literature

For general background, see: Deisenhofer & Michel (1989 ▶); Pan *et al.* (2006 ▶); Su & Xu (2004 ▶). For a related structure, see: Zhang *et al.* (2008 ▶). For the thickness of the aromatic ring, see: Cotton & Wilkinson (1972 ▶).
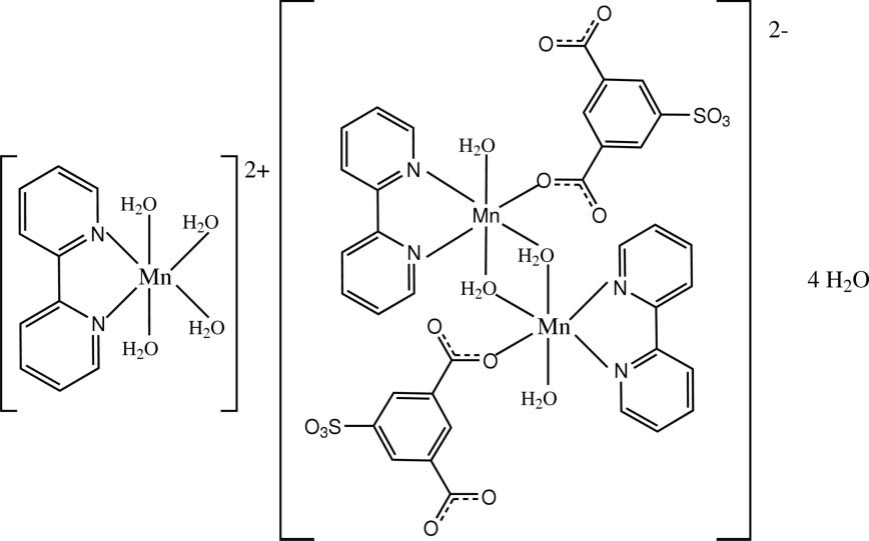


## Experimental

### 

#### Crystal data

[Mn(C_10_H_8_N_2_)(H_2_O)_4_][Mn_2_(C_8_H_3_O_7_S)_2_(C_10_H_8_N_2_)_2_(H_2_O)_4_]·4H_2_O
*M* *_r_* = 1335.89
Monoclinic, 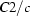

*a* = 19.656 (4) Å
*b* = 9.1286 (17) Å
*c* = 32.035 (6) Å
β = 96.584 (7)°
*V* = 5710.1 (18) Å^3^
*Z* = 4
Mo *K*α radiation
μ = 0.82 mm^−1^
*T* = 295 (2) K
0.40 × 0.36 × 0.20 mm

#### Data collection

Rigaku R-AXIS RAPID IP diffractometer
Absorption correction: multi-scan (*ABSCOR*; Higashi, 1995 ▶) *T*_min_ = 0.742, *T*_max_ = 0.850
32384 measured reflections
6179 independent reflections
4071 reflections with *I* > 2σ(*I*)
*R*_int_ = 0.071

#### Refinement

*R*[*F* ^2^ > 2σ(*F* ^2^)] = 0.047
*wR*(*F* ^2^) = 0.123
*S* = 1.09
6179 reflections
375 parameters
H-atom parameters constrained
Δρ_max_ = 0.96 e Å^−3^
Δρ_min_ = −0.41 e Å^−3^

### 

Data collection: *PROCESS-AUTO* (Rigaku, 1998 ▶); cell refinement: *PROCESS-AUTO*; data reduction: *CrystalStructure* (Rigaku/MSC, 2002 ▶); program(s) used to solve structure: *SIR92* (Altomare *et al.*, 1993 ▶); program(s) used to refine structure: *SHELXL97* (Sheldrick, 2008 ▶); molecular graphics: *ORTEP-3 for Windows* (Farrugia, 1997 ▶); software used to prepare material for publication: *WinGX* (Farrugia, 1999 ▶).

## Supplementary Material

Crystal structure: contains datablocks I, global. DOI: 10.1107/S1600536808020540/ng2471sup1.cif

Structure factors: contains datablocks I. DOI: 10.1107/S1600536808020540/ng2471Isup2.hkl

Additional supplementary materials:  crystallographic information; 3D view; checkCIF report

## Figures and Tables

**Table 1:** Selected bond lengths (Å)

Mn1—N1	2.253 (3)
Mn1—N2	2.212 (3)
Mn1—O1	2.116 (2)
Mn1—O1*W*	2.278 (2)
Mn1—O1*W*^i^	2.305 (2)
Mn1—O2*W*	2.161 (2)
Mn2—N3	2.282 (3)
Mn2—O3*W*	2.145 (2)
Mn2—O4*W*	2.1707 (19)

Symmetry code: (i) 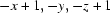.

**Table 2:** Hydrogen-bond geometry (Å, °)

*D*—H⋯*A*	*D*—H	H⋯*A*	*D*⋯*A*	*D*—H⋯*A*
O1*W*—H1*A*⋯O5*W*	0.85	1.87	2.701 (3)	164
O1*W*—H1*B*⋯O2	0.93	1.62	2.548 (3)	173
O2*W*—H2*A*⋯O3^ii^	0.85	1.87	2.716 (3)	170
O2*W*—H2*B*⋯O6^iii^	0.89	1.91	2.789 (3)	167
O3*W*—H3*A*⋯O5^iv^	0.90	1.85	2.746 (3)	176
O3*W*—H3*B*⋯O4^ii^	0.95	1.68	2.621 (3)	170
O4*W*—H4*A*⋯O3^v^	0.95	1.82	2.727 (3)	157
O4*W*—H4*B*⋯O7^iv^	0.89	1.90	2.780 (3)	168
O5*W*—H5*A*⋯O7^iii^	0.95	1.83	2.770 (4)	170
O5*W*—H5*B*⋯O6*W*^i^	0.95	2.04	2.749 (5)	131
O6*W*—H6*A*⋯O6^vi^	0.91	2.26	3.128 (5)	160
O6*W*—H6*B*⋯O3	0.92	2.27	3.109 (5)	151

Symmetry codes: (i) 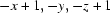; (ii) 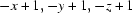; (iii) 
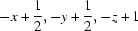
; (iv) 
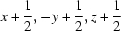
; (v) 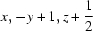; (vi) 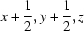.

**Fig. 1.**
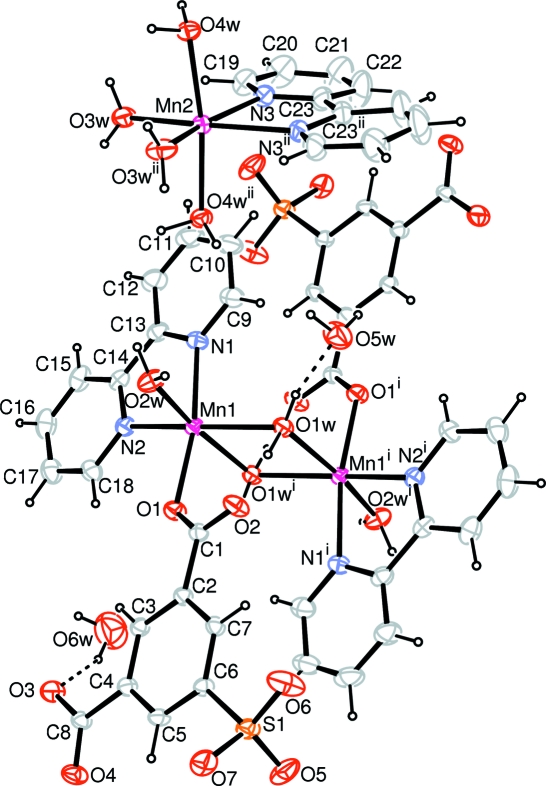
The molecular structure of the title compound with 30% probability displacement (arbitrary spheres for H atoms). Dashed lines indicate hydrogen bonding [symmetry codes: (i) 1 - x, -y, 1 - z; (ii) 1 - x, y, 3/2 - z].

**Fig. 2.**
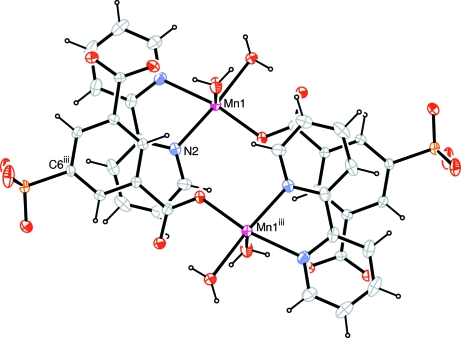
A diagram showing π-π stacking between aromatic rings [symmetry code: (iii) 1 - x, 1 - y, 1 - z].

## supplementary crystallographic information

### Comment

As π-π stacking between aromatic rings plays an important role in electron transfer process in some biological system (Deisenhofer & Michel, 1989), π-π stacking has attracted our much attention in past years (Su & Xu, 2004; Pan *et al.*, 2006). In order to investigate the influence of substituents of the aromatic compounds on stacking between parallel aromatic rings, the title Mn^II^ compound incorporating sulfoisophthalate ligand has recently been prepared and its crystal structure is reported here.

The crystal structure of the title compound consists of dimeric Mn^II^ complex anions, monomeric Mn^II^ complex cations and lattice water molecules (Fig. 1).

The complex anion is located across on an inversion center, two independent parts are bridged by two water molecules with approximately identical Mn—O(bridge) bond distances (Table 1) and a normal Mn—O—Mn^i^ bond angle of 103.85 (8)° [symmetry code: (i) 1 - *x*, -*y*, 1 - *z*]. Each Mn^II^ ion is coordinated by one 2,2'-bipyridine (bipy) ligand, one sulfoisophtalate anion, two bridge water molecules and one terminal water molecule in a distorted octahedral geometry. The Mn—O(bridge) bond distances are significantly longer than Mn—O(terminal) bond distances. In the Mn_2_O_2_ core the Mn···Mn and O···O distances are 3.6071 (11) and 2.826 (4) Å, respectively. The benzene ring of sulfoisophthalate and bipy ring system coordinated to the same Mn^II^ ion are nearly co-planar, the dihedral angle being 2.62 (14)°.

The complex cation has twofold rotation symmetry, with the Mn2 and the mid-point of the C23—C23^II^ bond located on the twofold rotation axis [symmetry code: (ii) 1 - *x*, *y*, 3/2 - *z*]. The Mn2 ion is coordinated by four water molecules and chelated by one bipy in a distorted octahedral geometry.

Partially overlapped arrangement between nearly parallel [dihedral angle 3.49 (19)°] bipy and benzene ring of sulfoisophthalate of the adjacent complex anion is observed in the crystal structure (Fig. 2). The perpendicular distance of the centroid of the N2-pyridine ring on the C6^iii^-benzene ring is 3.357 Å, and the perpendicular distance of the centroid of the C6^iii^-benzene ring on the N2-pyridine ring is 3.425 Å, they are significantly shorter than the van der Waals thickness of the aromatic ring (Cotton & Wilkinson, 1972) and indicate the existence of π-π stacking involving sulfoisophthalate anion, similar to that found in a related Co^II^ complex with sulfoisophthalate ligand (Zhang *et al.*, 2008).

The extensive O—H···O and C—H···O hydrogen bonding network presents in the crystal structure (Table 2), which helps to stabilize the crystal structure.

### Experimental

The monosodium 5-sulfoisophthalate (0.27 g, 1 mmol), sodium carbonate (0.11 g, 1 mmol), 2,2'-bipyridine (0.16 g, 1 mmol), manganese chloride tetrahydrate (0.20 g, 1 mmol), water (8 ml) and ethanol (2 ml) were sealed in a 20-ml Teflon-lined, stainless-steel autoclave. The autoclave was heated to 398 K for 36 h and then cooled to room temperature over 24 h. The solution was filtered and the single crystals of the title compound were obtained from the filtrate after 10 d.

### Refinement

Water H atoms were located in a difference Fourier map and refined as riding in as-found relative positions with *U*_iso_(H) = 1.5*U*_eq_(O). Aromatic H atoms were placed in calculated positions with C—H = 0.93 Å and refined in riding mode with *U*_iso_(H) = 1.2*U*_eq_(C).

### Crystal data

**Table:**

[Mn(C_10_H_8_N_2_)(H_2_O)_4_][Mn_2_(C_8_H_3_O_7_S)_2_(C_10_H_8_N_2_)_2_(H_2_O)_4_]·4H_2_O	*F*_000_ = 2748
*M**_r_* = 1335.89	*D*_x_ = 1.554 Mg m^−^^3^
Monoclinic, *C*2/*c*	Mo *K*α radiation λ = 0.71073 Å
Hall symbol: -C 2yc	Cell parameters from 8728 reflections
*a* = 19.656 (4) Å	θ = 2.2–24.5º
*b* = 9.1286 (17) Å	µ = 0.82 mm^−^^1^
*c* = 32.035 (6) Å	*T* = 295 (2) K
β = 96.584 (7)º	Plate, yellow
*V* = 5710.1 (18) Å^3^	0.40 × 0.36 × 0.20 mm
*Z* = 4	

### Data collection

**Table:**

Rigaku R-AXIS RAPID IP diffractometer	6179 independent reflections
Radiation source: fine-focus sealed tube	4071 reflections with *I* > 2σ(*I*)
Monochromator: graphite	*R*_int_ = 0.071
Detector resolution: 10.0 pixels mm^-1^	θ_max_ = 27.0º
*T* = 295(2) K	θ_min_ = 2.1º
ω scans	*h* = −24→23
Absorption correction: multi-scan(ABSCOR; Higashi, 1995)	*k* = −11→11
*T*_min_ = 0.742, *T*_max_ = 0.850	*l* = −40→40
32384 measured reflections	

### Refinement

**Table:**

Refinement on *F*^2^	Secondary atom site location: difference Fourier map
Least-squares matrix: full	Hydrogen site location: inferred from neighbouring sites
*R*[*F*^2^ > 2σ(*F*^2^)] = 0.047	H-atom parameters constrained
*wR*(*F*^2^) = 0.123	*w* = 1/[σ^2^(*F*_o_^2^) + (0.0531*P*)^2^ + 1.8129*P*] where *P* = (*F*_o_^2^ + 2*F*_c_^2^)/3
*S* = 1.09	(Δ/σ)_max_ = 0.001
6179 reflections	Δρ_max_ = 0.97 e Å^−^^3^
375 parameters	Δρ_min_ = −0.41 e Å^−^^3^
Primary atom site location: structure-invariant direct methods	Extinction correction: none

### Special details

**Table:**

Geometry. All e.s.d.'s (except the e.s.d. in the dihedral angle between two l.s. planes) are estimated using the full covariance matrix. The cell e.s.d.'s are taken into account individually in the estimation of e.s.d.'s in distances, angles and torsion angles; correlations between e.s.d.'s in cell parameters are only used when they are defined by crystal symmetry. An approximate (isotropic) treatment of cell e.s.d.'s is used for estimating e.s.d.'s involving l.s. planes.
Refinement. Refinement of *F*^2^ against ALL reflections. The weighted *R*-factor *wR* and goodness of fit *S* are based on *F*^2^, conventional *R*-factors *R* are based on *F*, with *F* set to zero for negative *F*^2^. The threshold expression of *F*^2^ > σ(*F*^2^) is used only for calculating *R*-factors(gt) *etc*. and is not relevant to the choice of reflections for refinement. *R*-factors based on *F*^2^ are statistically about twice as large as those based on *F*, and *R*- factors based on ALL data will be even larger.

### Fractional atomic coordinates and isotropic or equivalent isotropic displacement parameters (Å^2^)

**Table:**

	*x*	*y*	*z*	*U*_iso_*/*U*_eq_	
Mn1	0.52324 (2)	0.17373 (5)	0.524402 (13)	0.03219 (14)	
Mn2	0.5000	0.11071 (7)	0.7500	0.03035 (18)	
S1	0.17116 (4)	0.28491 (9)	0.36332 (2)	0.0385 (2)	
N1	0.60400 (14)	0.1056 (3)	0.57642 (7)	0.0367 (6)	
N2	0.61544 (13)	0.3064 (3)	0.51746 (8)	0.0357 (6)	
N3	0.56862 (14)	−0.0915 (3)	0.75678 (8)	0.0390 (6)	
O1	0.46005 (11)	0.2725 (2)	0.47425 (6)	0.0380 (5)	
O2	0.36256 (11)	0.1687 (2)	0.48870 (7)	0.0441 (6)	
O3	0.45078 (12)	0.6278 (3)	0.35348 (7)	0.0494 (6)	
O4	0.35555 (11)	0.6315 (3)	0.30833 (6)	0.0458 (6)	
O5	0.17450 (13)	0.2000 (3)	0.32526 (8)	0.0640 (8)	
O6	0.14849 (13)	0.1983 (4)	0.39683 (9)	0.0785 (10)	
O7	0.13200 (11)	0.4188 (3)	0.35423 (7)	0.0495 (6)	
O1W	0.44697 (10)	−0.0134 (2)	0.52604 (6)	0.0350 (5)	
H1A	0.4411	−0.0471	0.5501	0.052*	
H1B	0.4137	0.0501	0.5136	0.052*	
O2W	0.47816 (11)	0.2970 (3)	0.57186 (7)	0.0479 (6)	
H2A	0.5042	0.3135	0.5946	0.072*	
H2B	0.4371	0.2837	0.5806	0.072*	
O3W	0.58479 (12)	0.2600 (3)	0.75394 (6)	0.0511 (7)	
H3A	0.6123	0.2751	0.7779	0.077*	
H3B	0.6103	0.3029	0.7338	0.077*	
O4W	0.49868 (11)	0.1280 (2)	0.81748 (6)	0.0368 (5)	
H4A	0.4857	0.2251	0.8238	0.055*	
H4B	0.5394	0.1176	0.8327	0.055*	
O5W	0.40641 (16)	−0.1425 (3)	0.59492 (8)	0.0703 (8)	
H5A	0.3967	−0.0709	0.6149	0.105*	
H5B	0.4381	−0.2183	0.6027	0.105*	
O6W	0.5729 (2)	0.4097 (4)	0.36611 (13)	0.1194 (13)	
H6A	0.6043	0.4786	0.3758	0.179*	
H6B	0.5384	0.4658	0.3524	0.179*	
C1	0.39638 (16)	0.2497 (3)	0.46703 (8)	0.0308 (7)	
C2	0.35851 (15)	0.3205 (3)	0.42861 (8)	0.0291 (6)	
C3	0.39081 (16)	0.4194 (3)	0.40419 (8)	0.0306 (7)	
H3	0.4362	0.4450	0.4122	0.037*	
C4	0.35618 (15)	0.4800 (3)	0.36818 (8)	0.0311 (7)	
C5	0.28809 (16)	0.4397 (3)	0.35622 (9)	0.0337 (7)	
H5	0.2643	0.4794	0.3321	0.040*	
C6	0.25587 (15)	0.3399 (3)	0.38050 (8)	0.0291 (6)	
C7	0.29045 (15)	0.2815 (3)	0.41659 (9)	0.0311 (7)	
H7	0.2684	0.2164	0.4329	0.037*	
C8	0.39040 (17)	0.5872 (3)	0.34085 (9)	0.0340 (7)	
C9	0.59329 (19)	0.0111 (4)	0.60721 (10)	0.0471 (9)	
H9	0.5513	−0.0367	0.6058	0.056*	
C10	0.6419 (2)	−0.0177 (4)	0.64070 (11)	0.0588 (11)	
H10	0.6327	−0.0828	0.6617	0.071*	
C11	0.7038 (2)	0.0510 (5)	0.64246 (12)	0.0635 (12)	
H11	0.7378	0.0317	0.6644	0.076*	
C12	0.7155 (2)	0.1487 (4)	0.61154 (11)	0.0555 (10)	
H12	0.7574	0.1966	0.6126	0.067*	
C13	0.66478 (17)	0.1759 (4)	0.57867 (9)	0.0383 (8)	
C14	0.67202 (16)	0.2851 (3)	0.54492 (9)	0.0361 (7)	
C15	0.73194 (18)	0.3614 (4)	0.54134 (12)	0.0479 (9)	
H15	0.7706	0.3448	0.5603	0.057*	
C16	0.7340 (2)	0.4622 (4)	0.50942 (13)	0.0569 (10)	
H16	0.7736	0.5155	0.5070	0.068*	
C17	0.6765 (2)	0.4829 (4)	0.48121 (12)	0.0519 (9)	
H17	0.6768	0.5489	0.4591	0.062*	
C18	0.61831 (19)	0.4036 (4)	0.48648 (10)	0.0453 (8)	
H18	0.5793	0.4185	0.4676	0.054*	
C19	0.6369 (2)	−0.0855 (4)	0.76152 (11)	0.0535 (10)	
H19	0.6576	0.0059	0.7651	0.064*	
C20	0.6783 (2)	−0.2072 (5)	0.76141 (15)	0.0759 (13)	
H20	0.7258	−0.1990	0.7653	0.091*	
C21	0.6470 (3)	−0.3405 (5)	0.75534 (18)	0.0976 (18)	
H21	0.6730	−0.4251	0.7542	0.117*	
C22	0.5767 (3)	−0.3485 (5)	0.75088 (16)	0.0856 (15)	
H22	0.5551	−0.4390	0.7471	0.103*	
C23	0.53805 (18)	−0.2222 (4)	0.75198 (11)	0.0481 (9)	

### Atomic displacement parameters (Å^2^)

**Table:**

	*U*^11^	*U*^22^	*U*^33^	*U*^12^	*U*^13^	*U*^23^
Mn1	0.0267 (3)	0.0370 (3)	0.0316 (2)	−0.0038 (2)	−0.00182 (19)	0.00509 (19)
Mn2	0.0315 (4)	0.0302 (3)	0.0292 (3)	0.000	0.0031 (3)	0.000
S1	0.0252 (5)	0.0501 (5)	0.0383 (4)	−0.0054 (4)	−0.0040 (3)	0.0078 (4)
N1	0.0360 (17)	0.0400 (15)	0.0335 (13)	0.0056 (13)	0.0015 (12)	0.0044 (11)
N2	0.0313 (16)	0.0383 (15)	0.0367 (14)	−0.0040 (12)	0.0003 (12)	0.0006 (11)
N3	0.0380 (18)	0.0372 (16)	0.0403 (14)	0.0053 (13)	−0.0016 (12)	−0.0037 (12)
O1	0.0245 (13)	0.0469 (13)	0.0404 (12)	−0.0055 (10)	−0.0051 (10)	0.0116 (10)
O2	0.0263 (13)	0.0559 (14)	0.0490 (13)	−0.0031 (11)	0.0002 (10)	0.0247 (11)
O3	0.0418 (15)	0.0621 (15)	0.0428 (12)	−0.0237 (12)	−0.0021 (11)	0.0143 (11)
O4	0.0379 (14)	0.0610 (15)	0.0378 (12)	−0.0096 (11)	0.0011 (10)	0.0182 (11)
O5	0.0496 (17)	0.0700 (18)	0.0650 (16)	0.0079 (14)	−0.0254 (13)	−0.0247 (14)
O6	0.0302 (15)	0.128 (3)	0.0738 (18)	−0.0267 (16)	−0.0079 (13)	0.0576 (18)
O7	0.0284 (14)	0.0553 (15)	0.0616 (15)	0.0062 (11)	−0.0093 (11)	−0.0033 (12)
O1W	0.0324 (13)	0.0365 (12)	0.0355 (11)	−0.0016 (10)	0.0011 (9)	0.0079 (9)
O2W	0.0325 (14)	0.0698 (17)	0.0408 (12)	−0.0003 (12)	0.0010 (10)	−0.0106 (11)
O3W	0.0553 (17)	0.0643 (16)	0.0326 (11)	−0.0299 (13)	0.0005 (11)	0.0056 (11)
O4W	0.0344 (13)	0.0441 (13)	0.0310 (10)	0.0056 (10)	−0.0006 (9)	−0.0009 (9)
O5W	0.104 (2)	0.0568 (16)	0.0541 (15)	0.0119 (16)	0.0275 (16)	0.0038 (12)
O6W	0.113 (3)	0.105 (3)	0.139 (3)	−0.016 (2)	0.010 (2)	0.022 (2)
C1	0.0299 (19)	0.0316 (16)	0.0299 (15)	0.0009 (14)	0.0000 (13)	0.0020 (12)
C2	0.0247 (17)	0.0316 (16)	0.0306 (14)	−0.0001 (13)	0.0008 (12)	0.0031 (12)
C3	0.0256 (17)	0.0344 (16)	0.0312 (15)	−0.0035 (13)	0.0001 (12)	0.0011 (12)
C4	0.0285 (18)	0.0361 (17)	0.0291 (15)	−0.0032 (13)	0.0044 (13)	0.0024 (12)
C5	0.0297 (19)	0.0428 (18)	0.0272 (14)	0.0005 (14)	−0.0023 (13)	0.0056 (13)
C6	0.0210 (16)	0.0363 (16)	0.0293 (14)	−0.0010 (13)	0.0007 (12)	0.0011 (12)
C7	0.0285 (18)	0.0319 (16)	0.0329 (15)	−0.0038 (13)	0.0029 (13)	0.0052 (12)
C8	0.035 (2)	0.0394 (18)	0.0280 (15)	−0.0066 (15)	0.0036 (14)	0.0077 (13)
C9	0.054 (2)	0.051 (2)	0.0368 (18)	0.0071 (18)	0.0062 (16)	0.0033 (15)
C10	0.083 (3)	0.055 (2)	0.0367 (19)	0.023 (2)	−0.001 (2)	0.0066 (16)
C11	0.069 (3)	0.067 (3)	0.048 (2)	0.025 (2)	−0.021 (2)	−0.003 (2)
C12	0.044 (2)	0.061 (2)	0.057 (2)	0.0091 (19)	−0.0145 (18)	−0.0003 (19)
C13	0.034 (2)	0.0420 (18)	0.0368 (16)	0.0072 (15)	−0.0032 (14)	−0.0067 (14)
C14	0.0298 (19)	0.0402 (18)	0.0376 (16)	0.0011 (14)	0.0015 (14)	−0.0100 (13)
C15	0.029 (2)	0.049 (2)	0.065 (2)	−0.0034 (16)	0.0048 (17)	−0.0138 (18)
C16	0.044 (3)	0.047 (2)	0.083 (3)	−0.0121 (18)	0.023 (2)	−0.011 (2)
C17	0.055 (3)	0.046 (2)	0.058 (2)	−0.0078 (18)	0.019 (2)	0.0038 (17)
C18	0.049 (2)	0.0446 (19)	0.0428 (18)	−0.0084 (17)	0.0074 (16)	0.0079 (15)
C19	0.045 (2)	0.051 (2)	0.063 (2)	0.0116 (19)	−0.0021 (18)	−0.0091 (18)
C20	0.049 (3)	0.079 (3)	0.097 (3)	0.024 (2)	−0.002 (2)	−0.019 (3)
C21	0.083 (4)	0.060 (3)	0.143 (5)	0.035 (3)	−0.016 (3)	−0.030 (3)
C22	0.085 (4)	0.038 (2)	0.129 (4)	0.013 (2)	−0.010 (3)	−0.017 (2)
C23	0.055 (2)	0.0366 (19)	0.050 (2)	0.0049 (17)	−0.0065 (19)	−0.0032 (15)

### Geometric parameters (Å, °)

**Table:**

Mn1—N1	2.253 (3)	O6W—H6B	0.9184
Mn1—N2	2.212 (3)	C1—C2	1.509 (4)
Mn1—O1	2.116 (2)	C2—C3	1.394 (4)
Mn1—O1W	2.278 (2)	C2—C7	1.395 (4)
Mn1—O1W^i^	2.305 (2)	C3—C4	1.386 (4)
Mn1—O2W	2.161 (2)	C3—H3	0.9300
Mn2—N3^ii^	2.282 (3)	C4—C5	1.398 (4)
Mn2—N3	2.282 (3)	C4—C8	1.521 (4)
Mn2—O3W	2.145 (2)	C5—C6	1.396 (4)
Mn2—O3W^ii^	2.145 (2)	C5—H5	0.9300
Mn2—O4W^ii^	2.1707 (19)	C6—C7	1.379 (4)
Mn2—O4W	2.1707 (19)	C7—H7	0.9300
S1—O6	1.444 (2)	C9—C10	1.378 (5)
S1—O5	1.452 (3)	C9—H9	0.9300
S1—O7	1.456 (2)	C10—C11	1.364 (6)
S1—C6	1.765 (3)	C10—H10	0.9300
N1—C9	1.345 (4)	C11—C12	1.372 (5)
N1—C13	1.351 (4)	C11—H11	0.9300
N2—C18	1.337 (4)	C12—C13	1.387 (5)
N2—C14	1.351 (4)	C12—H12	0.9300
N3—C19	1.334 (4)	C13—C14	1.490 (4)
N3—C23	1.337 (4)	C14—C15	1.384 (4)
O1—C1	1.263 (3)	C15—C16	1.380 (5)
O2—C1	1.255 (3)	C15—H15	0.9300
O3—C8	1.265 (4)	C16—C17	1.377 (5)
O4—C8	1.247 (3)	C16—H16	0.9300
O1W—Mn1^i^	2.305 (2)	C17—C18	1.380 (5)
O1W—H1A	0.8508	C17—H17	0.9300
O1W—H1B	0.9294	C18—H18	0.9300
O2W—H2A	0.8554	C19—C20	1.378 (5)
O2W—H2B	0.8930	C19—H19	0.9300
O3W—H3A	0.8964	C20—C21	1.368 (6)
O3W—H3B	0.9466	C20—H20	0.9300
O4W—H4A	0.9507	C21—C22	1.375 (7)
O4W—H4B	0.8932	C21—H21	0.9300
O5W—H5A	0.9496	C22—C23	1.383 (5)
O5W—H5B	0.9445	C22—H22	0.9300
O6W—H6A	0.9118	C23—C23^ii^	1.486 (7)
			
O1—Mn1—O2W	93.44 (9)	C3—C2—C7	119.6 (3)
O1—Mn1—N2	96.13 (9)	C3—C2—C1	121.4 (3)
O2W—Mn1—N2	101.09 (9)	C7—C2—C1	118.9 (2)
O1—Mn1—N1	168.94 (9)	C4—C3—C2	120.9 (3)
O2W—Mn1—N1	86.24 (9)	C4—C3—H3	119.5
N2—Mn1—N1	73.13 (9)	C2—C3—H3	119.5
O1—Mn1—O1W	90.36 (8)	C3—C4—C5	119.1 (3)
O2W—Mn1—O1W	92.89 (8)	C3—C4—C8	121.9 (3)
N2—Mn1—O1W	164.17 (8)	C5—C4—C8	119.0 (3)
N1—Mn1—O1W	100.70 (9)	C6—C5—C4	120.0 (3)
O1—Mn1—O1W^i^	84.97 (8)	C6—C5—H5	120.0
O2W—Mn1—O1W^i^	168.90 (8)	C4—C5—H5	120.0
N2—Mn1—O1W^i^	90.01 (8)	C7—C6—C5	120.5 (3)
N1—Mn1—O1W^i^	97.42 (8)	C7—C6—S1	120.6 (2)
O1W—Mn1—O1W^i^	76.15 (8)	C5—C6—S1	118.9 (2)
O3W—Mn2—O3W^ii^	101.14 (14)	C6—C7—C2	119.9 (3)
O3W—Mn2—O4W^ii^	85.10 (8)	C6—C7—H7	120.1
O3W^ii^—Mn2—O4W^ii^	89.62 (8)	C2—C7—H7	120.1
O3W—Mn2—O4W	89.62 (8)	O4—C8—O3	125.3 (3)
O3W^ii^—Mn2—O4W	85.10 (8)	O4—C8—C4	116.9 (3)
O4W^ii^—Mn2—O4W	171.68 (12)	O3—C8—C4	117.7 (3)
O3W—Mn2—N3^ii^	165.06 (10)	N1—C9—C10	122.7 (4)
O3W^ii^—Mn2—N3^ii^	93.52 (10)	N1—C9—H9	118.6
O4W^ii^—Mn2—N3^ii^	92.20 (8)	C10—C9—H9	118.6
O4W—Mn2—N3^ii^	94.54 (9)	C11—C10—C9	118.7 (4)
O3W—Mn2—N3	93.52 (10)	C11—C10—H10	120.7
O3W^ii^—Mn2—N3	165.06 (10)	C9—C10—H10	120.7
O4W^ii^—Mn2—N3	94.54 (8)	C10—C11—C12	119.5 (3)
O4W—Mn2—N3	92.20 (8)	C10—C11—H11	120.3
N3^ii^—Mn2—N3	72.01 (14)	C12—C11—H11	120.3
O6—S1—O5	112.31 (19)	C11—C12—C13	119.9 (4)
O6—S1—O7	114.02 (17)	C11—C12—H12	120.0
O5—S1—O7	110.72 (14)	C13—C12—H12	120.0
O6—S1—C6	106.50 (14)	N1—C13—C12	120.6 (3)
O5—S1—C6	106.36 (14)	N1—C13—C14	115.9 (3)
O7—S1—C6	106.37 (14)	C12—C13—C14	123.4 (3)
C9—N1—C13	118.5 (3)	N2—C14—C15	121.2 (3)
C9—N1—Mn1	124.3 (2)	N2—C14—C13	115.4 (3)
C13—N1—Mn1	116.87 (19)	C15—C14—C13	123.4 (3)
C18—N2—C14	118.5 (3)	C16—C15—C14	119.6 (3)
C18—N2—Mn1	122.9 (2)	C16—C15—H15	120.2
C14—N2—Mn1	118.6 (2)	C14—C15—H15	120.2
C19—N3—C23	118.9 (3)	C17—C16—C15	119.1 (3)
C19—N3—Mn2	123.6 (2)	C17—C16—H16	120.4
C23—N3—Mn2	117.3 (2)	C15—C16—H16	120.4
C1—O1—Mn1	123.47 (18)	C16—C17—C18	118.5 (3)
Mn1—O1W—Mn1^i^	103.85 (8)	C16—C17—H17	120.8
Mn1—O1W—H1A	116.7	C18—C17—H17	120.8
Mn1^i^—O1W—H1A	118.6	N2—C18—C17	123.0 (3)
Mn1—O1W—H1B	87.4	N2—C18—H18	118.5
Mn1^i^—O1W—H1B	108.5	C17—C18—H18	118.5
H1A—O1W—H1B	117.0	N3—C19—C20	123.6 (4)
Mn1—O2W—H2A	116.0	N3—C19—H19	118.2
Mn1—O2W—H2B	127.5	C20—C19—H19	118.2
H2A—O2W—H2B	103.2	C21—C20—C19	117.4 (4)
Mn2—O3W—H3A	122.4	C21—C20—H20	121.3
Mn2—O3W—H3B	133.7	C19—C20—H20	121.3
H3A—O3W—H3B	101.9	C20—C21—C22	119.6 (4)
Mn2—O4W—H4A	108.2	C20—C21—H21	120.2
Mn2—O4W—H4B	115.0	C22—C21—H21	120.2
H4A—O4W—H4B	103.2	C21—C22—C23	120.0 (4)
H5A—O5W—H5B	120.3	C21—C22—H22	120.0
H6A—O6W—H6B	102.3	C23—C22—H22	120.0
O2—C1—O1	124.9 (3)	N3—C23—C22	120.4 (4)
O2—C1—C2	117.4 (3)	N3—C23—C23^ii^	116.40 (19)
O1—C1—C2	117.7 (2)	C22—C23—C23^ii^	123.2 (3)

Symmetry codes: (i) −*x*+1, −*y*, −*z*+1; (ii) −*x*+1, *y*, −*z*+3/2.

### Hydrogen-bond geometry (Å, °)

**Table:**

*D*—H···*A*	*D*—H	H···*A*	*D*···*A*	*D*—H···*A*
O1W—H1A···O5W	0.85	1.87	2.701 (3)	164
O1W—H1B···O2	0.93	1.62	2.548 (3)	173
O2W—H2A···O3^iii^	0.85	1.87	2.716 (3)	170
O2W—H2B···O6^iv^	0.89	1.91	2.789 (3)	167
O3W—H3A···O5^v^	0.90	1.85	2.746 (3)	176
O3W—H3B···O4^iii^	0.95	1.68	2.621 (3)	170
O4W—H4A···O3^vi^	0.95	1.82	2.727 (3)	157
O4W—H4B···O7^v^	0.89	1.90	2.780 (3)	168
O5W—H5A···O7^iv^	0.95	1.83	2.770 (4)	170
O5W—H5B···O6W^i^	0.95	2.04	2.749 (5)	131
O6W—H6A···O6^vii^	0.91	2.26	3.128 (5)	160
O6W—H6B···O3	0.92	2.27	3.109 (5)	151
C16—H16···O2^vii^	0.93	2.36	3.281 (4)	169
C17—H17···O6^vii^	0.93	2.43	3.337 (5)	166

Symmetry codes: (iii) −*x*+1, −*y*+1, −*z*+1; (iv) −*x*+1/2, −*y*+1/2, −*z*+1; (v) *x*+1/2, −*y*+1/2, *z*+1/2; (vi) *x*, −*y*+1, *z*+1/2; (i) −*x*+1, −*y*, −*z*+1; (vii) *x*+1/2, *y*+1/2, *z*.

## References

[bb1] Altomare, A., Cascarano, G., Giacovazzo, C. & Guagliardi, A. (1993). *J. Appl. Cryst.* **26**, 343–350.

[bb2] Cotton, F. A. & Wilkinson, G. (1972). *Advances in Inorganic Chemistry*, p. 120. New York: John Wiley & Sons.

[bb3] Deisenhofer, J. & Michel, H. (1989). *EMBO J.* **8**, 2149–2170. 10.1002/j.1460-2075.1989.tb08338.x PMC401143 2676514

[bb4] Farrugia, L. J. (1997). *J. Appl. Cryst.* **30**, 565.

[bb5] Farrugia, L. J. (1999). *J. Appl. Cryst.* **32**, 837–838.

[bb6] Higashi, T. (1995). *ABSCOR* Rigaku Corporation, Tokyo, Japan.

[bb8] Pan, T.-T., Su, J.-R. & Xu, D.-J. (2006). *Acta Cryst.* E**62**, m2183–m2185.

[bb9] Rigaku (1998). *PROCESS-AUTO* Rigaku Corporation, Tokyo, Japan.

[bb10] Rigaku/MSC (2002). *CrystalStructure* Rigaku/MSC, The Woodlands, Texas, USA.

[bb11] Sheldrick, G. M. (2008). *Acta Cryst.* A**64**, 112–122. 10.1107/S0108767307043930 18156677

[bb12] Su, J.-R. & Xu, D.-J. (2004). *J. Coord. Chem.* **57**, 223–229.

[bb13] Zhang, B.-Y., Nie, J.-J. & Xu, D.-J. (2008). *Acta Cryst.* E**64**, m986. 10.1107/S1600536808019843 PMC2961914 21203083

